# Scope and Limits of Teriparatide Use in Delayed and Nonunions: A Case Series

**DOI:** 10.3390/clinpract11010009

**Published:** 2021-01-29

**Authors:** Petros Ismailidis, Norbert Suhm, Martin Clauss, Annegret Mündermann, Dieter Cadosch

**Affiliations:** 1 Department of Orthopaedics and Traumatology, University Hospital Basel, Spitalstrasse 21, 4031 Basel, Switzerland; norbert.suhm@usb.ch (N.S.); martin.clauss@usb.ch (M.C.); annegret.muendermann@unibas.ch (A.M.); dcadosch@gmx.net (D.C.); 2 Department of Biomedical Engineering, University of Basel, Gewerbestrasse 14, 4123 Allschwil, Switzerland; 3 Department of Clinical Research, University of Basel, Schanzenstrasse 55, 4056 Basel, Switzerland; 4 Center for Musculosceletal Infections, University Hospital Basel, Spitalstrasse 21, 4031 Basel, Switzerland

**Keywords:** teriparatide, delayed union, nonunion, fracture healing, pseudarthrosis

## Abstract

Nonunion is known to occur in up to 10% of all bone fractures. Until recently, the treatment options considered in cases of delayed union and nonunion focused on revision surgery and improvement of local healing. Lately, teriparatide has been introduced as an osteoanabolic factor that induces fracture healing in cases with delayed or nonunions. We report on a series of five cases of delayed and nonunions treated with teriparatide: delayed unions of an atypical femoral fracture, of a multifragmentary clavicle fracture, and of a periprosthetic humeral fracture; nonunion of a tibial and fibular fracture; and infected nonunion of a tibial and fibular fracture. Based on this series, the indications and limits of application of teriparatide in cases of impaired fracture healing are discussed. Due to the “off-label” character of this application, informed consent, and cost coverage from the healthcare insurance must be obtained prior to treatment. In our experience and according to the limited existing literature, teriparatide is a safe feasible treatment in cases of delayed and nonunions with a reasonable need of resources. While adequate biomechanical stability remains the cornerstone of fracture healing, as well as healing of nonunions, teriparatide could help avoid repetitive surgeries, especially in atrophic delayed and nonunions, as well as in patients with impaired fracture healing undergoing bisphosphonate therapy. There is an urgent need for widely accepted definitions, standardized protocols, as well as further clinical trials in the field of impaired fracture healing.

## 1. Introduction

Avoiding and treating impaired fracture healing remain topics of extensive discussion in the orthopaedic field [1,2,3,4]. To date, a widely accepted definition of nonunions and delayed unions is lacking. For nonunions, the most commonly used definition refers to fractures of at least 9 months since injury without any visible signs of progressive healing for 3 months (American Food and Drug Administration (FDA)), whereas other studies have stated a broader definition of nonunions as a disturbance of normal healing with the expectation that no consolidation will be achieved without focused and accurate treatment [5]. Definitions regarding delayed unions are even less clear. In most cases, delayed unions refer to fractures without a progress in healing within a reasonable timetable considering the fracture type and patient characteristics, because the timeframe can differ greatly between cases. While the exact prevalence of nonunions in fracture of the extremities is not known, several studies reported a prevalence from 5 to 10% [3,4,6].

Various methods have been discussed for the treatment of nonunions and preventing a delayed union evolving into a nonunion. Modern research efforts are oriented into bone callus enhancement by avoiding risk factors [1] as well as improving local mechanical and biological factors [4,7]. Furthermore, cellular therapies, biophysical stimulation of fracture healing, as well as systemic pharmacological therapy (bisphosphonates, denosumab, strontium ranelate, teriparatide) aimed at improving the fracture healing process have been reported [7,8]. Recently, the use of teriparatide as an osteoanabolic factor, inducing fracture healing in the treatment of nonunions or delayed unions, has been introduced. A systematic review in 2020 identified 20 articles (15 case reports, 4 case series, 1 prospective study) reporting on a total of 64 patients [2], and emphasized the need for further data on the use of teriparatide in nonunions and delayed unions.

The objective of this case series was to report our first experience with the use of teriparatide in nonunions and delayed unions, and to discuss the indications, potential, and limitations of this treatment. 

## 2. Case Series

Five patients with nonunions and delayed unions were included in this case series. Nonunion was defined according to the FDA definition as a fracture with a minimum of 9 months since injury without any visible progressive signs of healing for 3 months. Delayed union was defined as a fracture at least 3 months since injury or stabilization without any signs of clinical or radiological healing. Written patient consent was received from all patients and archived. The study was approved by the regional ethics committee.

At the presence of a nonunion or delayed union, patients were initially screened for risk factors, namely diabetes, anaemia, medication (NSAID, steroids), alcohol consumption, and smoking [1]. If possible, risk factors were corrected. Patients received appropriate daily living advice and support. Following patient information and formal informed consent, pharmacological treatment included off label application of once daily subcutaneous injections of 20 µg teriparatide. The injections in the thigh or abdomen, according to the patient’s preference, were performed by the patients themselves or with help from healthcare assistants using the manufacturer’s device. The health insurance provider was contacted to secure a cost coverage for this off-label therapy. In case of antiresorptive therapies for osteoporosis, these were stopped prior to teriparatide therapy. Vitamin D was supplemented with 800 IU daily, at the presence of Vitamin D insufficiency (serum 25(OH)D < < 75 nmol/L) [9]. In case of inadequate calcium daily intake (<1200 mg/d) it was supplemented [10].

Patients were clinically and radiologically monitored regularly in our outpatient clinic. Our standard monitoring involved a 6 week; 12 week; 6 month and 1 year follow-up with further follow-ups if needed, until adequate clinical and radiological verification of bone union could be confirmed. Clinical signs of bone union were the absence of pain or tenderness at the fracture site at rest or on palpation with ability to weight-bear [6]. Radiologic signs of bone union were evidence of bridging of the fracture site by callus, trabeculae, or bone as well as bridging of the fracture site at three out of four cortices assessed by both anteroposterior and lateral plain radiographs [6,11].

Amino-terminal propeptide of type I procollagen (P1NP) and/or Beta-CrossLaps (Beta-CTx) in serum were monitored throughout the treatment with teriparatide, as biological response markers [12]. Teriparatide associated complications, treatment duration until union, and need of revision surgery were documented.

**Figure 1:**
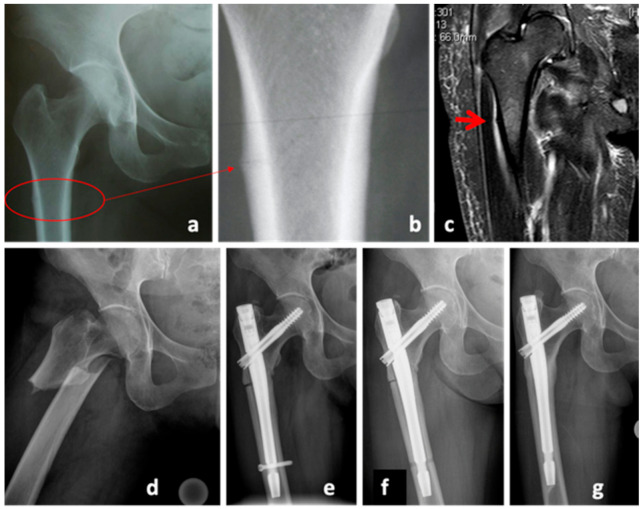
Case 1, X-Rays and MRI of the femur on the day of initial presentation (**a**–**c**) and one day later (**d**), showing the diagnosis of an atypical femoral fracture (AFF) under bisphosphonate therapy. X-ray of the hip, 7 months after trauma in the presence of a delayed union (**e**), after nail dynamization (**f**), and at the end of the teriparatide therapy (**g**), 31 months after trauma.

Case 1:

A 51-year-old female Asian patient (body mass 47.7 kg; height: 150 cm; body mass index (BMI): 20.9 kg/m2) was treated with bisphosphonates for osteoporosis for the last 14 years. She presented with acute pain at the right thigh without previous trauma. The radiological examinations included x-rays as well as MRI, both initially evaluated as normal. The following day, the patient presented with an atypical femoral fracture (AFF) (Figure 1d). In retrospect, there was a bone stress reaction as a prodromal sign of an AFF (AFF, Figure 1a–c). Closed reduction and osteosynthesis of the proximal femur was performed (Gamma3 Trochanteric Nail, Stryker, MI, USA). Initially, the treatment with bisphosphonates was continued. In hindsight, this could have prevented the fracture union, however the Task Force of the American Society for Bone and Mineral Research Guidelines [13] were not yet published at that time and, therefore, there was no clear indication for discontinuing the bisphosphonate therapy. Seven months later, the patient presented with a delayed union with persistent pain and lack of radiologic signs of union; she was referred for further osteological treatment with the diagnosis of an atrophic delayed union (Figure 1e). The biomechanical evaluation showed a varus malalignment. The decision was made to furthermore delay revision surgery with fracture reduction, and instead remove the distal screw to allow dynamization of the nail. The antiresorptive therapy was stopped, a vitamin D insufficiency supplemented and the treatment with teriparatide initiated (Figure 1f). The total duration of teriparatide therapy was 24 months. Gradual callus build-up as well as pain reduction was noted in further follow-ups. After completion of the therapy, the patient was pain-free and radiological signs of bony union were present (Figure 1g). 

**Figure 2:**
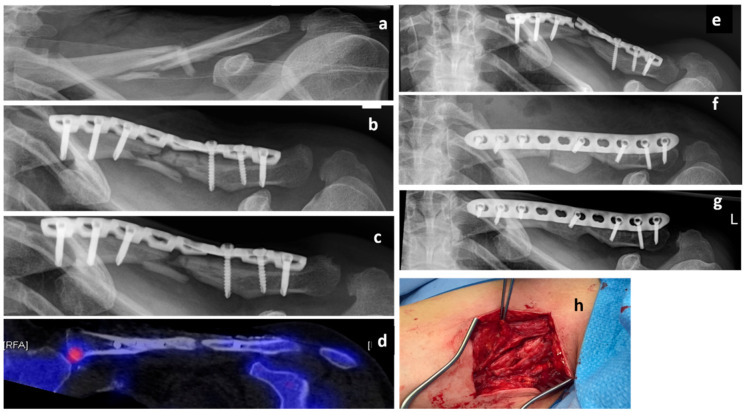
Case 2, x-rays presenting the initial clavicle fracture (**a**), after open reduction and internal fixation (ORIF) (**b**), and in the presence of a delayed union 6 months after trauma (c). A single-photon emission computed tomography (SPECT-CT) scan showing a lack of biological activity at the fracture zone 17 months after trauma (**d**). X-rays of implant failure 18 months after trauma (**e**), postoperative x-ray after revision osteosynthesis with tricortical iliac bone graft (**f**), as well as final result 2 years after trauma (**g**). Intraoperative photographs and showing the atrophic nonunion (**h**).

Case 2:

A 50-year-old female Caucasian patient (body mass 66 kg; height: 165 cm; BMI: 24.2 kg/m^2^) presented with a multifragmentary fracture of the left clavicle (AO 15.2C, non-dominant arm) after a bicycle accident (Figure 2a). Due to fracture comminution and shortening of the clavicle of more than 2 cm, an operative treatment was indicated. The patient was treated with an open reduction and internal fixation (ORIF) and plate osteosynthesis (LCP Reconstruction Plate 3.5, DePuy Synthes, USA) (Figure 2b). Serial X-rays during the first 6 months showed no radiological signs of union and the patient was diagnosed with an atrophic delayed union. She was referred for osteological treatment (Figure 2c). Due to inadequate Vitamin D levels and low calcium intake, both were supplemented. There were no further predisposing factors for nonunion. The radiological analysis showed a lack of contact between the bony fragments (Figure 2c. However, the patient was pain-free and, therefore, the decision against further surgery was made. A treatment with teriparatide was initiated. Subsequent biomarker analyses revealed a 61% increase in P1NP and a 208% increase in Beta CT-x after 3 months of treatment. However, there were no radiological signs of union. A single-photon emission computed tomography (SPECT-CT) after 11 months of teriparatide treatment showed no biological activity at the fracture zone (Figure 2d). Consequently, the patient was informed about the high risk of implant failure and low chance of fracture healing. Due to the fact that she was still pain-free and showed no signs of relevant impairment in the activities of daily living, she decided against an operation. As expected, the patient presented with plate failure 18 months after the initial surgery (Figure 2e). The plate was removed, and an ORIF with autologous tricortical iliac bone graft (LCP 3.5 mm Plate, DePuy Synthes, USA) was conducted (Figure 2f). The teriparatide treatment was continued. The further course was uneventful and clinical and radiological bone union was achieved 6 months postoperatively (Figure 2g).

**Figure 3:**
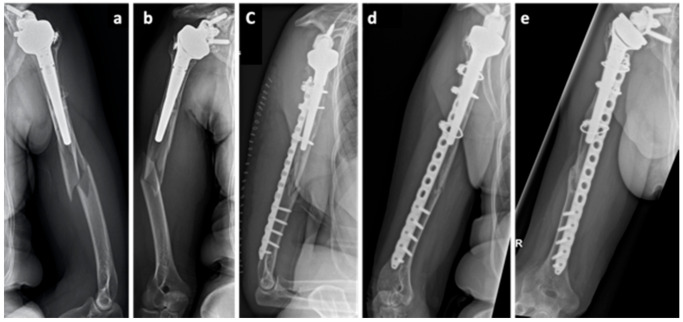
Case 3, x-ray showing the initial periprosthetic humeral fracture (UCS I.1B1) (**a**,**b**), the initial postoperative radiograph (**c**), delayed union 6 months after trauma (**d**) and bone union at the end of the teriparatide treatment, 12 months after trauma (**e**).

Case 3:

A 64-year-old Caucasian female patient (body mass 58 kg; height: 159 cm; BMI: 24.2 kg/m^2^) presented with a periprosthetic humeral fracture (UCS I.1B1) [14] due to a bicycle accident (Figure 3a,b). Operative treatment was conducted with an angular stable plate and cerclage osteosynthesis (Figure 3c). Six months postoperatively, the patient had persisting pain at the fracture site and lack of radiological signs of union (Figure 3d). She was referred to our clinic with the diagnosis of delayed union. In the absence of alternative surgical options, conservative treatment was started. This included vitamin D and calcium supplementation as well as administration of teriparatide. A long-term NSAID intake was discontinued. Subsequent follow-ups and serial x-rays showed a progressive build-up of callus. The P1NP values increased to double the values documented at the beginning of the therapy. Clinical and radiological bone union was achieved 12 months postoperatively.

**Figure 4:**
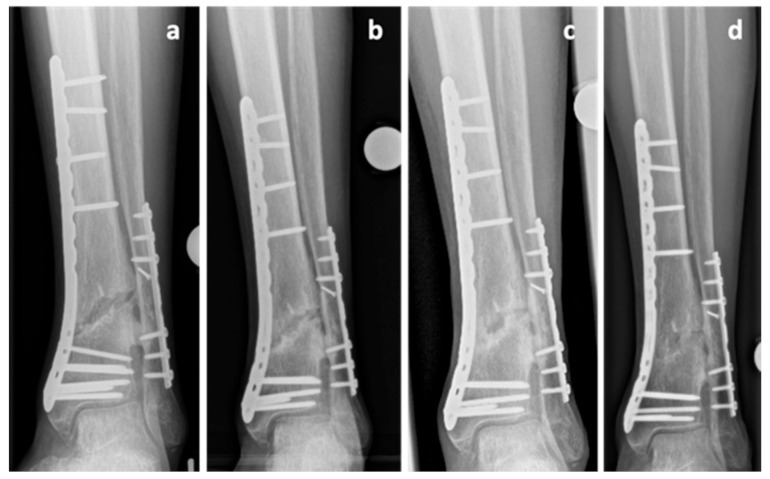
Case 4, x-rays showing a nonunion 29 months after the initial injury at the initiation of the teriparatide therapy (**a**). Serial x-rays after 3 and 15 months of teriparatide treatment showing gradual callus build-up (**b**,**c**). Radiological and clinical union was achieved 4 years after the initial injury (**d**).

Case 4:

A 77-year-old Caucasian male patient (body mass: 77 kg; height: 178 cm; BMI: 24.3 kg/m^2^) presented with a grade 2 [15] open fracture of the femur (AO 33 C1) and a closed tibial and fibular fracture (AO 43 A3) following a bicycle accident (collision against a truck). He was treated with an ORIF and plate osteosynthesis of the femur (less invasive stabilization system (LISS) plate, DePuy Synthes, USA) as well as ORIF and plate osteosynthesis of the tibia (distal tibia LCP plate, DePuy Synthes, USA) and fibula (1/3 tubular plate, DePuy Synthes, USA). Eighteen months later an atrophic nonunion was diagnosed and revision surgery of both femur (LISS pate) and tibia (distal tibia LCP plate) was performed. This procedure included decortication, re-osteosynthesis, and application of autologous cancellous bone from the iliac crest. Intraoperative samples showed no sign of infection. The fracture of the femur showed normal healing. Once again, the tibial fracture showed no healing tendency and the diagnosis of an atrophic nonunion (Figure 4a) was made 11 months after the revision surgery operation and 29 months after the initial injury. The patient was referred for further osteological treatment of the tibia. Biomechanical analysis showed a correct anatomical reduction and therefore the decision against further operations was made. Calcium and vitamin D supplementation was initiated and a teriparatide treatment was conducted over a period of 21 months. CTX increased by 83% after the teriparatide therapy. The fracture gap showed gradual callus build-up on the serial x rays (Figure 4b,c). A radiological and clinical union was achieved 4 years after the initial injury (Figure 4d).

**Figure 5:**
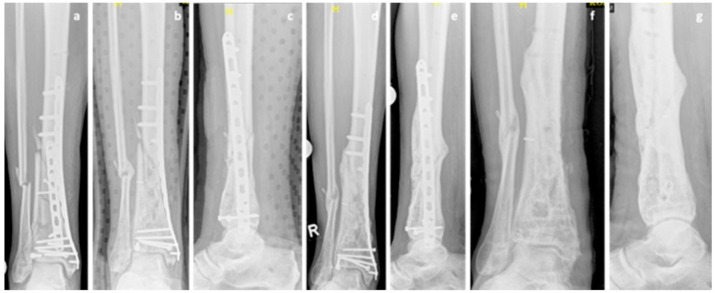
Case 5, x-rays showing the postoperative outcome after initial treatment of grade 1 open tibial and fibular fracture (**a**), after partial hardware removal (anterior tibial plate) and autologous cancellous bone grafting at the presence of an atrophic tibia nonunion 9 months after trauma (**b**,**c**) and after achieving bone union 18 months after the initial injury (**d**,**e**). Final result after hardware removal 24 months after trauma (**f**,**g**).

Case 5:

A 67-year-old Caucasian male patient (body mass 87 kg; height: 182 cm; BMI: 26.3 kg/m^2^) presented with a grade 1 open fracture of the right tibia and fibula (AO 43B2.2) and a grade 2 open fracture of the left tibia and fibula (AO 43B2.2) after jumping off a burning bus. He was initially treated with external fixators, and a few days later, ORIF and double-plate osteosynthesis (distal tibia LCP Plate, 3.5 LCP plate, single crews, DePuy Synthes, USA) on both sides (Figure 5a) were performed. While the left side showed regular bone healing, the radiological imaging of the right side revealed no signs of bone healing in serial radiographs. These findings were accompanied by persistent pain and the patient was diagnosed with an atrophic delayed union of the tibia, four months after the injury. NSAID medication was stopped, treatment of diabetes mellitus was optimized, and the decision to start teriparatide treatment was made. However, no progress in bone healing was observed. The double plate osteosynthesis was considered to be too rigid and, therefore, compromising fracture healing. Revision surgery was performed at the presence of an atrophic nonunion 9 months postoperatively with removal of the anterior tibial plate and autologous cancellous bone grafting (Figure 5b,c). Initially, there was no suspicion of an infection and therefore the original medial osteosynthesis was left in situ, no extensive debridement was performed. The intraoperative biopsies showed an osteomyelitis with peptostreptococcus species. Antibiotic treatment was initiated and continued as suppressive therapy. The teriparatide therapy was continued until signs of clinical healing and radiologic signs of bone union were achieved 18 months after the initial injury (Figure 5d,e). P1NP increased by 100% and CTX increased by 82% during teriparatide therapy indicating increased bone activity. The hardware was removed 24 months after the injury (Figure 5f). The intraoperative biopsies taken at the time of the hardware removal were negative, and the antibiotic therapy was discontinued.

## 3. Discussion

This case series illustrates some possibilities for the application of teriparatide in treating nonunions and delayed unions. However, based on these cases, we cannot judge if and to what extent fracture healing in the presented cases was due to teriparatide application. Nonetheless, according to our experience, the use of teriparatide in nonunions and delayed unions is feasible and does not lead to adverse effects. Providing proper patient instruction, it can be a powerful aid in avoiding repetitive surgery without excessive use of resources.

### 3.1. Safety and Feasibility of Teriparatide Application

Teriparatide treatment can be challenging. The patient must be instructed in performing daily, subcutaneous injections and regularly monitored for possible side effects, such as serum creatinine increase [16,17], headache, dizziness, hyperuricemia, leg cramps [18], allergic events, nausea, and injection site reaction [19]. In this cases series, all patients were able to administer the subcutaneous injections at home, either by themselves or with the help of healthcare assistants. No adverse events were reported. This is in accordance with the review of Canintika [2], who concluded that teriparatide application in nonunions and delayed unions is safe. Although daily injection of 20 µg teriparatide is the standard dosage, weekly injections have also been reported and could be a feasible application in cases where patients are not able to administer the daily subcutaneous injections [20,21,22,23]. Of the studies reporting on teriparatide usage for the treatment of nonunions, four chose to administer a weekly SC injection of 56.5 µg [20,21,22,23]. In this case series, we did not have administration problems and, therefore, chose to administer the standard dosage of 20 µg daily. The fact that the off-label usage requires a cost coverage from the health insurance provider, did not seem to be a problem in our healthcare system. This might be explained by the far lower costs of teriparatide treatment, compared to other therapeutic options, entailing mainly revision surgery.

### 3.2. Spectrum of Teriparatide Indications in Nonunions and Delayed Unions

To date, twenty articles (15 case reports, 4 case series, 1 prospective study) reported on the use of teriparatide in nonunions and/or delayed unions. Several fracture types were included in these studies. While the majority of the cases involved long bone fractures (femoral and tibial fractures being the most common) teriparatide was additionally used for vertebral fractures [24,25,26,27] and in a case of a sternal fracture [28]. Current literature suggests that there is no reason to believe that teriparatide should only be effective in specific bones. The fact that nonunions are most common after long bone fractures, is probably responsible for the more frequent usage of teriparatide for nonunions of these bones. According to the principles of nonunion management [29], a hypertrophic nonunion occurs as a consequence of lacking mechanical stability and not because of impaired biological bone healing potential. Therefore, in the presence of callus formation, improving the mechanical stability should be the critical step in achieving bone union [29]. Contrary to that, atrophic nonunions with minimal or no callus requires stimulation of the bone healing activity. We believe that teriparatide application should primarily be reserved for atrophic nonunions, as illustrated in cases 2, 3, and 4. Yet, the beneficial effect of teriparatide cannot overcome major biomechanical and local biological problems, such as the absence of fragment contact or the presence of deperiosted bone fragments, as seen in case 2. Lastly, the infected nonunion belongs to a different entity and subcategory of impaired bone healing. As indicated in case 5, the expectation to achieve bone healing through teriparatide treatment alone does not seem realistic in the presence of an untreated infection. The principles of debridement of infected tissues, bone grafting, and antibiotic treatment remain the cornerstone of treating infected nonunions [30].

Regarding the success rates of teriparatide application for delayed and nonunions, any statements should be made with great caution. The literature consists mainly of case reports and case series’. Therefore there is a great potential of unsuccessful treatments being underrepresented in the literature, since authors would be less likely to publish a case report of a failed treatment. That being said, the existing literature suggests a high union rate of 95.3% [2]. The necessary duration of teriparatide treatment until a bone union is achieved varies greatly, from as low as two months [26] to 9 months [31]. The ideal treatment duration depends on the fracture type and the response to the therapy, and should be decided in an individual basis. Nevertheless, since the usage of teriparatide for osteoporosis is only approved for a total treatment duration, limited to 24 months, it seems reasonable to apply this limit in cases of usage of this drug for the treatment on nonunions as well. 

### 3.3. Nonunions and Delayed Unions with Bisphosphonate Treatment

Bisphosphonates have been established in the treatment of osteoporosis and are currently the most commonly prescribed medication for osteoporosis [32]. However, concerns have been raised that they may lead to an oversuppression of bone turnover and, therefore, contribute to a delayed union [32]. In the presence of a nonunion, changing a bisphosphonate therapy to teriparatide therapy is reasonable. Especially in the case of patients with AFFs under bisphosphonate therapy (case 1), discontinuation of bisphosphonates and treatment with teriparatide is indicated. This treatment recommendation has been included in the Guidelines of the American Society for Bone and Mineral Research ASBMR [33] for “those fractures that appear not to heal on conservative therapy”. If indicated, bisphosphonate therapy may be resumed after complete fracture healing. Whether antiresorptive treatment after stopping teriparatide could help maintain the viability of the improved fracture healing remains unclear.

### 3.4. Lack of Definitions for Nonunions and Delayed Unions as Well as of Standardized Protocols

Clearly, there is a lack of evidence regarding the use of teriparatide in nonunions and delayed unions. To date, only limited evidence originating mainly from case reports and case series is available [2]. Furthermore, the absence of an accepted definition of nonunion and delayed union makes the comparison among studies very difficult. In the review by Canintika et al. [2] on teriparatide treatment of nonunions, the definition of nonunion and the time of initiation of treatment with teriparatide after fracture varied largely between different studies. For instance, Bednar et al. [25] reported of a nonunion and initiated the teriparatide treatment already three months after an odontoid fracture. Conversely, Kastirr et al. [34], in the larger series published to date, reported on 32 patients with a delayed or nonunion with a mean fracture to treatment time of 24.3 ± 17.8 months. 

Lastly, qualitative evidence can only be produced if nonunions and delayed unions are treated in a standardized setting, according to predefined protocols. In our case series, although we tried to standardize the procedures of clinical, radiological, and laboratory monitoring of the patients, this was not always possible, partly because of the complexity of the cases and the referral from external institutions.

## 4. Conclusions

Based on the presented case series and our experience, teriparatide is a safe, feasible treatment in cases of nonunions and delayed unions with reasonable need of resources. While avoiding risk factors, along with improving mechanical and biological factors remains the cornerstone of fracture healing, teriparatide can aid in avoiding repetitive surgeries, especially in atrophic nonunions and delayed unions, as well as in patients with impaired fracture healing under bisphosphonate therapy. These results clearly emphasize the urgent need for widely accepted definitions, standardized protocols, as well as further clinical trials in the field of impaired fracture healing.

## Data Availability

The data presented in this study are available on request from the corresponding author.
